# Retraction Note: Long Non-coding RNA MALAT1/microRNA-143/VEGFA Signal Axis Modulates Vascular Endothelial Injury-Induced Intracranial Aneurysm

**DOI:** 10.1186/s11671-023-03828-2

**Published:** 2023-03-20

**Authors:** Ge Gao, Yang Zhang, Jian Yu, Yu Chen, Daqun Gu, Chaoshi Niu, Xianming Fu, Jianjun Wei

**Affiliations:** 1 Department of Neurosurgery, The First Affiliated Hospital of USTC, Division of Life Sciences and Medicine, University of Science and Technology of China, 17 Lu’ Jiang Road, Hefei, 230001 Anhui People’s Republic of China; 2 Department of Neurosurgery, Anhui Provincial Hospital Affiliated to Anhui Medical University, Hefei, Anhui People’s Republic of China

**Retraction note: Nanoscale Research Letters (2020) 15:139** **https://doi.org/10.1186/s11671-020-03357-2**

The Editors in Chief have retracted this article. After publication, concerns were raised around a seeming partial overlap within images presented in Fig. 1C as well as in multiple panels in Figs. 2C, D, E. The authors provided partial data, but were unable to provide uncropped, original files. The Editors in Chief, therefore, have lost confidence in the integrity of the article's findings. None of the authors agree to this retraction.

